# SOG – Spezielle Orthopädische Geriatrie: Erste Zwischenergebnisse einer randomisierten kontrollierten Studie zur integrierten orthogeriatrischen Versorgung in der elektiven Hüft- und Kniegelenkendoprothetik

**DOI:** 10.1007/s00132-023-04466-3

**Published:** 2024-01-18

**Authors:** Matthias Meyer, Katrin Michalk, Felix Greimel, Günther Maderbacher, Joachim Grifka, Tobias Kappenschneider

**Affiliations:** Orthopädische Klinik für die Universität Regensburg, Asklepios Klinikum Bad Abbach, Kaiser-Karl-V.-Allee 3, 93077 Bad Abbach, Deutschland

**Keywords:** Postoperative Mobilität, Postoperative Komplikationen, Patient reported outcome measure, Hüfttotalendoprothese, Knietotalendoprothese, Postoperative mobility, Postoperative complications, Patient reported outcome measure, Total hip replacement, Total knee replacement

## Abstract

**Hintergrund:**

Für die Versorgung geriatrischer Patienten bei elektiven orthopädischen Operationen existieren in Deutschland bisher noch keine adäquaten Versorgungskonzepte. Die SOG-Studie evaluiert hierzulande erstmalig den Einfluss eines komplexen orthogeriatrischen Co-Managements auf das Outcome älterer Patienten mit elektivem Hüft- und Kniegelenkersatz im Vergleich zur orthopädischen Standardversorgung.

**Methodik:**

In die Zwischenanalyse der noch laufenden Studie wurden 174 Patienten eingeschlossen, wovon 87 Patienten in die Interventionsgruppe und 87 Patienten in die Kontrollgruppe randomisiert wurden. Das SOG-Versorgungsmodell besteht aus Screening, präoperativem Assessment mit präoperativer Intervention/Optimierung, Operation nach dem Fast-Track-Prinzip und multimodaler perioperativer Versorgung im orthogeriatrischen Team. Die Kontrollgruppe erhielt eine orthopädische Standardbehandlung. Verglichen wurden Mobilität, Komplikationen und „patient-reported outcome measures“ (PROM).

**Ergebnisse:**

Die Interventionsgruppe wies postoperativ gegenüber der Kontrollgruppe zu allen Erfassungszeitpunkten eine klinisch relevant verbesserte Mobilität auf (*p* < 0,01). Die Komplikationsauswertung zeigte eine signifikante Risikoreduktion für Minor-Komplikationen (*p* < 0,01) sowie einen deutlichen Trend zur Risikoreduktion für Major-Komplikationen. Die Auswertung der PROM zeigte unabhängig von der Intervention eine signifikante Verbesserung der Gelenkfunktion und der allgemeinen gesundheitsbedingten Lebensqualität.

**Schlussfolgerung:**

Integrierte orthogeriatrische Modelle, wie die Spezielle Orthopädische Geriatrie, könnten zukünftig die Versorgung geriatrischer Patienten in der elektiven orthopädischen Chirurgie verbessern und vor allem sicherer gestalten.

**Graphic abstract:**

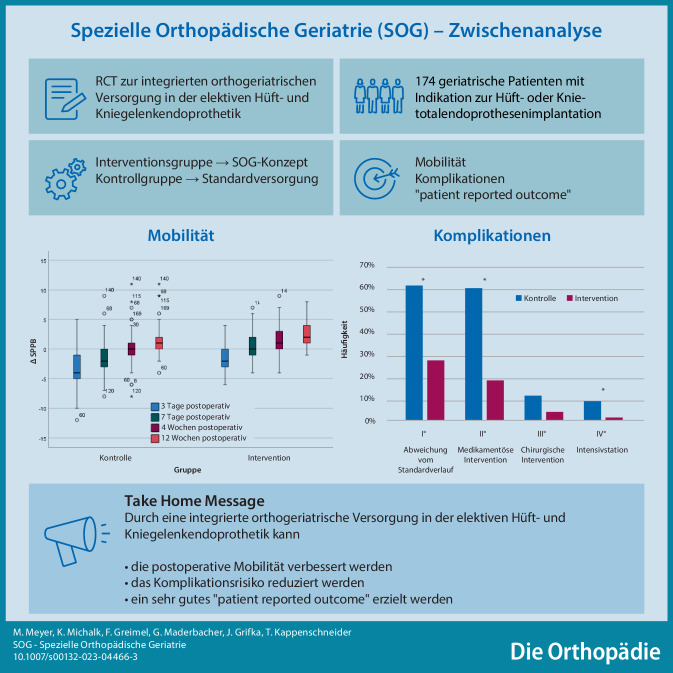

## Abstract (en)

**Background:**

To date, there are no adequate care concepts for geriatric patients undergoing elective orthopaedic surgery in Germany. The Special Orthopaedic Geriatrics (SOG) study evaluates for the first time in Germany the impact of comprehensive orthogeriatric co-management on the outcome of elderly patients with elective hip and knee replacements compared to standard orthopaedic care.

**Methods:**

The interim analysis of the ongoing study included 174 patients; 87 patients were randomized to the intervention group and 87 to the control group. The SOG care model consists of screening, preoperative assessment with preoperative intervention, fast-track surgery and multimodal perioperative care in the orthogeriatric team. The control group received standard orthopaedic care. Mobility, complications, and patient-reported outcome measures after surgery were compared.

**Results:**

The SOG group showed a clinically relevant improvement in mobility postoperatively compared to the control group at all time points (*p* < 0.01). The evaluation of complications showed a significant risk reduction for minor complications (*p* < 0.01), as well as a clear trend towards a risk reduction for major complications. Patient-reported outcome measures showed a significant improvement in joint function and general health-related quality of life both in the SOG group and the control group.

**Conclusion:**

Integrated orthogeriatric care models such as SOG could improve the care of geriatric patients in elective orthopaedic surgery in the future and, above all, make it safer.

Die Versorgung älterer Patienten in der Orthopädie stellt eine zunehmende Herausforderung dar. Eine steigende Anzahl elektiv-orthopädischer Patienten ist nicht nur hochbetagt, sondern zeichnet sich durch Immobilität, Multimorbidität, Polypharmazie und Gebrechlichkeit (Frailty) aus. Die orthopädisch-chirurgische Standardversorgung gerät bei geriatrischen Patienten an ihre Grenzen. Das durch den G‑BA finanzierte Modellprojekt Spezielle Orthopädische Geriatrie (SOG) will die Versorgung geriatrischer Patienten in der elektiven orthopädischen Chirurgie verbessern.

Im Jahr 2060 werden etwa 30 % der Bevölkerung in Deutschland über 65 Jahre und ca. 11 % über 80 Jahre alt sein [2]. Infolgedessen wird auch die Anzahl geriatrischer Patienten zunehmen, die sich operativer Eingriffe unterziehen müssen. So wurde beispielsweise für die Bundesrepublik Deutschland eine Zunahme primärer Knietotalendoprothesen um 43 % bis 2050 prognostiziert [15]. Geriatrische Patienten gelten definitionsgemäß als vulnerabel und damit besonders anfällig für perioperative Komplikationen [18, 21, 22]. Um dieser Entwicklung zukünftig gerecht werden zu können, bedarf es neuer Versorgungskonzepte.

In der Alterstraumatologie erfolgt deshalb seit einigen Jahren sehr erfolgreich ein orthogeriatrisches Co-Management meist im Rahmen einer geriatrischen frührehabilitativen Komplexbehandlung (GFK). Hierbei wird der Patient bei Erfüllung bestimmter Strukturvoraussetzungen im multiprofessionellen Team postoperativ mitbehandelt. Einige Studien konnten zwischenzeitlich den Benefit einer solchen orthogeriatrischen Kooperation in der Alterstraumatologie zeigen. So resultiert ein orthogeriatrisches Co-Management hüftgelenksnaher Frakturen in einer Reduktion der Mortalität [29] und einer Verbesserung des funktionellen Outcomes [27].

Doch im Gegensatz zur Alterstraumatologie, in der überwiegend unfallchirurgische und somit notfallmäßige Eingriffe vorgenommen werden, existieren in reinen orthopädischen Fachabteilungen oder Fachkliniken mit schwerpunktmäßig elektiven Operationen bisher keine spezifischen orthogeriatrischen Versorgungsmodelle. Gerade hier bleibt das große Potenzial, den geriatrischen und oftmals multimorbiden Patienten bereits im Vorfeld optimal auf die Operation vorzubereiten, ihn vor der Operation altersmedizinisch vorzustellen und bereits präoperativ ein umfassendes geriatrisches Assessment zu erheben, bisher ungenutzt [19]. An der Klinik und Poliklinik für Orthopädie der Universität Regensburg am Asklepios Klinikum Bad Abbach wurde folglich das orthogeriatrische Versorgungsmodell „Spezielle Orthopädische Geriatrie (SOG)“ entwickelt, welches aktuell im Rahmen einer durch den Innovationsfonds des G‑BA geförderten, randomisierten kontrollierten Studie (RCT) bei geriatrischen Patienten mit elektivem Hüft- und Kniegelenkersatz evaluiert wird [13].

Ziel der Studie ist es, den Effekt einer integrierten orthogeriatrischen Versorgung im Vergleich zur rein orthopädischen Standardversorgung bei geriatrischen Patienten in der elektiven, primären Hüft- und Kniegelenkendoprothetik anhand multipler validierter Parameter zu evaluieren [13]. Primäre Zielgröße ist die Mobilität, gemessen anhand der Short Physical Performance Battery (SPPB) [7]. Die Studienhypothese lautet demzufolge, dass ein multimodales perioperatives orthogeriatrisches Co-Management bei elektiver Hüft- oder Knie-TEP-Implantation im Vergleich zur rein orthopädischen Standardversorgung in einer höheren postoperativen Mobilität der geriatrischen Patienten resultiert. Als sekundäre Outcomevariablen wurden peri- und postoperative Komplikationen sowie „patient-reported outcome measures“ (PROM) ausgewertet. Ein Gesamtüberblick über die vollständigen primären, sekundären und tertiären Zielparameter der noch laufenden SOG-Studie ist dem von Kappenschneider et al. publizierten Studienprotokoll zu entnehmen [13]. Mit dem Vorliegen der Endergebnisse ist voraussichtlich 2025 zu rechnen.

## Methodik

### Studiendesign

Die SOG-Studie ist eine monozentrische, prospektive, randomisierte, kontrollierte Interventionsstudie. Sie ist im Deutschen Register Klinischer Studien (DRKS) des Bundesinstituts für Arzneimittel und Medizinprodukte (BfArM) sowie auf der Registerplattform für internationale klinische Studien der Weltgesundheitsorganisation (WHO) unter der ID DRKS00024102 registriert. Projektträger der Studie ist das Deutsche Zentrum für Luft- und Raumfahrt (DLR), die Finanzierung erfolgt durch den Innovationsfond des G‑BA mit einer Fördersumme von ca. 1,4 Mio. € (Förderkennzeichen: 01VSF19030). Ein positives Votum der Ethikkommission der Universität Regensburg liegt vor (Nr. 20-1837-101). Konsortialpartner des Modellprojekts ist das Fachgebiet für Gesundheitsökonomie der Technischen Universität München.

Es erfolgte eine computergestützte 1:1 Randomisierung per „Randomizer“-Online-Tool (https://www.randomizer.at/, Randomizer Version 2.1.0, Institut für Medizinische Informatik, Statistik und Dokumentation, Medizinische Universität Graz, Österreich). Um eine ausgeglichene Zuteilungsreihenfolge zu gewährleisten, wurde eine stratifizierte permutierte Blockrandomisierung mit Stratifizierung nach Geschlecht (weiblich oder männlich) und Art der Versorgung (Knie oder Hüfte) verwendet. Die Studiendaten wurden präoperativ, an den postoperativen Tagen 1–7, 4 Wochen und 3 Monate nach Operation erhoben.

### Studienpopulation

**Figure 1:**
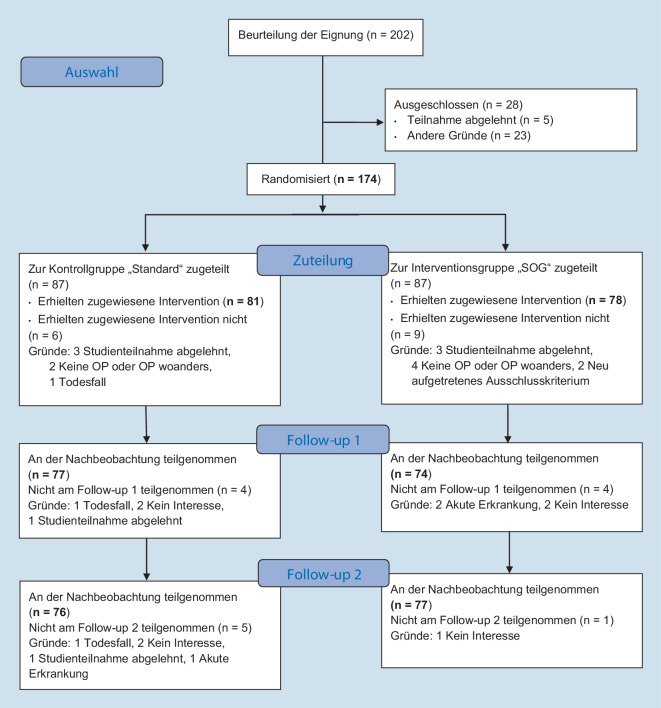

Folgende Einschlusskriterien wurden definiert: primäre Kox- oder Gonarthrose, Alter ≥ 70 Jahre + geriatrietypische Multimorbidität oder Alter ≥ 80 Jahre mit Indikation für elektiven Hüft- oder Kniegelenkersatz. Die Ausschlusskriterien waren: Alter < 70 Jahre, vorherige Fraktur/knöcherne Operation oder Tumor im Bereich des zu behandelnden Gelenks, akute Infektion und erhöhter Pflegebedarf (Pflegegrad ≥ 4). Die aktuelle Zwischenanalyse bezieht sich auf 174 Patienten aus dem Zeitraum von 01. April 2021 bis 30. September 2023, wovon nach Randomisierung 87 Probanden der Interventionsgruppe (SOG-Versorgungskonzept) und 87 Teilnehmer der Kontrollgruppe (orthopädische Standardbehandlung) zugewiesen wurden. Der weitere Patientenflow ist dem modifizierten CONSORT-Flussdiagramm (Abb. 1) zu entnehmen.

### Intervention (SOG-Versorgungsmodell) und Kontrollgruppe

Das SOG-Versorgungsprinzip vertritt einen ganzheitlichen Ansatz in der interdisziplinären Versorgung orthogeriatrischer Patienten mit elektiver Hüft‑/Knie-TEP und beinhaltet zusammenfassend fünf in sich ineinandergreifende Komponenten. Dies sind Screening, umfassendes präoperatives geriatrisches Assessment, präoperative Intervention [19], Operation nach dem Fast-Track-Prinzip und multimodale perioperative Versorgung im orthogeriatrischen Team. Die Patienten in der Kontrollgruppe erhalten eine orthopädische Standardversorgung. Bezüglich der Details zum Studienablauf mit Vorgehen in der Interventions- und Kontrollgruppe darf an dieser Stelle auf das bereits veröffentlichte Studienprotokoll verwiesen werden [13].

### Studienparameter

#### Short Physical Performance Battery (SPPB)

Die SPPB ist eine Testbatterie, welche die motorischen Kriterien Gleichgewicht, Ganggeschwindigkeit und Muskelkraft vereint. Sie beinhaltet die progressive Testung der statischen Balance (Side-by-Side‑, Semi-Tandem- und Tandem-Stand), die Messung der Ganggeschwindigkeit über 4 m und den Chair Rise Test (5-mal vom Stuhl aufstehen und Hinsetzen). Der Test ermöglicht sowohl einen Summenscore als auch einzelne Werte in den drei motorischen Dimensionen. Maximal sind 12 Punkte im Gesamtscore erreichbar [7].

#### Komplikationen nach Clavien-Dindo

Die Klassifikation nach Clavien-Dindo [5] dient der Erfassung und Kategorisierung postoperativer Komplikationen. Es existieren je nach erforderlicher Therapieform 5 Schweregrade:

- Grad I: Abweichung vom normalen postoperativen Verlauf mit definierten Behandlungsregimen
- Grad II: anderweitige Therapeutika/Bluttransfusionen
- Grad III: Einsatz von chirurgischen, endoskopischen oder radiologischen Interventionen
- Grad IV: lebensbedrohliche Komplikationen mit intensivmedizinischer Behandlung
- Grad V: Tod

#### Western Ontario and McMaster Universities Osteoarthritis Index (WOMAC)

Validierter Fragebogen zur Beurteilung von Patienten mit Kox‑/Gonarthrose. Er umfasst 24 Fragen, davon 5 zur Beurteilung von Schmerzen, zwei zur Gelenksteifheit und 17 zur körperlichen Funktion. Verwendet wurde die WOMAC-Likert-Version mit einer Skala von 0–4 Punkten für jede Frage. Bewertungsbereiche: Schmerz 0–20 Punkte, Steifheit 0–8 Punkte und körperliche Funktion 0–68 Punkte, wobei der höhere Wert das schlechtere Ergebnis bedeutet [18].

#### EQ-5D VAS

Komponente des EQ-5D-Gesundheitsfragebogens mit einer Visuellen Analogskala (VAS) mit einem Wertebereich von 0–100. Dabei werden die Befragten gebeten, ihren aktuellen Gesundheitszustand auf der Skala einzuschätzen [11].

### Statistik

Kontinuierliche Daten wurden als Mittelwert mit Standardabweichung, ordinalskalierte und nicht normalverteilte metrische Daten als Median mit Interquartilsabstand beschrieben. Kategorische Variablen wurden als absolute und relative Häufigkeiten angegeben. Vergleiche zwischen den beiden Behandlungsgruppen wurden dementsprechend mit dem Student’s t‑Test, dem Wilcoxon-Mann-Whitney-Test oder dem Chi-Quadrat-Test durchgeführt. Ein p‑Wert < 0,05 wurde bei allen Tests als statistisch signifikant angesehen. Alle Analysen wurden mit dem Statistical Package for the Social Sciences (IBM SPSS Statistics for Windows, Version 27.0, IBM Corp., Armonk, NY, USA) durchgeführt.

## Ergebnisse

Präoperativ zeigten sich weitgehend ausgeglichene Patientencharakteristika in Kontroll- und Interventionsgruppe (Tab. 1).

**Table 1:** 

Charakteristika	Gesamtkollektiv			Subgruppe Hüft-TEP			Subgruppe Knie-TEP		
	Standard (*n* = 87)	SOG (*n* = 87)	*p*-Wert	Standard (*n* = 52)	SOG (*n* = 48)	*p*-Wert	Standard (*n* = 35)	SOG (*n* = 39)	*p*-Wert
Frauen, *n* (%)	57 (65,5)	60 (69,0)	0,628	34 (65,4)	32 (66,7)	0,892	23 (65,7)	28 (71,8)	0,537
Hüft-TEP, *n* (%)	52 (59,8)	48 (55,2)	0,540	–	–	–	–	–	–
Alter (Jahre), MW ± SD	78,0 ± 4,7	79,2 ± 4,8	0,130	77,5 ± 4,6	79,1 ± 4,6	0,089	78,8 ± 4,8	79,2 ± 5,1	0,770
BMI (kg/m^2^), MW ± SD	29,3 ± 5,5	28,5 ± 4,5	0,273	29,0 ± 5,5	27,9 ± 4,9	0,318	29,9 ± 5,6	29,2 ± 3,8	0,542
Komorbiditäten insg., MW ± SD	7,7 ± 3,2	7,5 ± 3,2	0,712	7,4 ± 3,3	7,5 ± 3,4	0,890	8,1 ± 3,0	7,5 ± 2,9	0,397
CCI, Med (IQA)	5,0 (2)	5,0 (2)	0,380	5,0 (2)	5,0 (3)	0,062	5,0 (2)	5,0 (2)	0,308
Frailty nach Fried, Med (IQA)	2,0 (2)	3,0 (2)	0,313	2,0 (2)	3,0 (1)	0,090	2,0 (2)	2,0 (2)	0,821
Barthel, Med (IQA)	95,0 (10)	100,0 (10)	0,133	95,0 (10)	100,0 (15)	0,380	95,0 (10)	100,0 (10)	0,235
IADL, Med (IQA)	7,0 (2)	8,0 (2)	0,323	7,0 (2)	7,0 (2)	0,922	7,0 (2)	8,0 (1)	0,188
MMSE, Med (IQA)	27,0 (3)	27,0 (4)	0,414	27,5 (3)	27,0 (3)	0,521	27,0 (3)	26,0 (5)	0,641
GDS, Med (IQA)	2,0 (3)	3,0 (4)	0,537	3,0 (3)	3,0 (4)	0,579	2,0 (3)	3,0 (3)	0,698

*BMI* Body-Mass-Index, *CCI* Charlson-Komorbiditätsindex, *GDS* Geriatric Depression Scale, *IADL* Instrumental Activities of Daily Living, *IQA* Interquartilsabstand,* MMSE* Mini Mental Status Examination, *SOG* Spezielle Orthopädische Geriatrie

**Figure 2:**
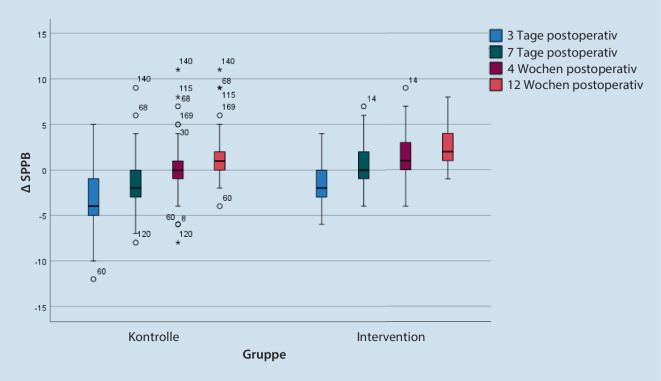

Die mediane SPPB nahm in der Kontroll- und Interventionsgruppe am 3. postoperativen Tag um 4 bzw. 2 Punkte ab. Während sich die SPPB in der Interventionsgruppe bereits nach einer Woche auf das Ausgangsniveau von 7 Punkten erholte, blieb die Kontrollgruppe mit 6 Punkten noch unter dem Ausgangsniveau zurück. Vier Wochen postoperativ erreichte die Kontrollgruppe mit 8 Punkten das Ausgangsniveau, während die Interventionsgruppe bereits eine Verbesserung auf 9 Punkte zeigte. Drei Monate postoperativ war die Mobilität in der Kontrollgruppe um einen Punkt, in der Interventionsgruppe um 3 Punkte besser als präoperativ. Die Unterschiede bzgl. der SPPB-Differenzen zum Ausgangswert in Kontroll- und Interventionsgruppe waren zu jedem Erfassungszeitpunkt sowohl für das Gesamtkollektiv als auch für die Subgruppen Hüft-TEP und Knie-TEP signifikant (*p* < 0,01; Gesamtkollektiv in Abb. 2). Die absoluten SPPB-Werte für Kontroll- und Interventionsgruppe sowie für die Subgruppen Hüft-TEP und Knie-TEP sind in Tab. 2 ersichtlich.

**Table 2:** 

Parameter	Gesamtkollektiv			Subgruppe Hüft-TEP			Subgruppe Knie-TEP		
	Kontrolle	SOG	*p*-Wert	Kontrolle	SOG	*p*-Wert	Kontrolle	SOG	*p*-Wert
SPPB, Med (IQA)									
Präoperativ	8,0 (4)	7,0 (4)	0,221	8,0 (6)	6,0 (4)	0,110	7,5 (3)	8,0 (3)	0,859
3. postoperativer Tag	4,0 (5)	6,0 (3)	**<** **0,001**	4,0 (3)	6,0 (4)	**0,032**	2,0 (4)	5,5 (3)	**<** **0,001**
7. postoperativer Tag	6,0 (4)	7,0 (4)	**<** **0,001**	6,5 (4)	8,0 (5)	**0,008**	5,0 (4)	7,0 (2)	**<** **0,001**
4 Wochen postoperativ	8,0 (5)	9,0 (4)	**0,041**	8,0 (4)	9,0 (5)	0,166	7,0 (4)	9,0 (4)	0,076
3 Monate postoperativ	9,0 (4)	10,0 (4)	0,120	9,0 (4)	9,0 (4)	0,729	8,0 (5)	10,0 (2)	**0,041**
Komplikationen, *n* (%)									
Clavien-Dindo I°	49 (60,5)	21 (26,9)	**<** **0,001**	32 (65,3)	9 (20,9)	**<** **0,001**	17 (53,1)	12 (34,3)	0,123
Clavien-Dindo II°	48 (59,3)	14 (17,9)	**<** **0,001**	32 (65,3)	7 (16,3)	**<** **0,001**	16 (50,0)	7 (20,0)	**0,010**
Clavien-Dindo III°	9 (11,1)	3 (3,8)	0,084	9 (18,4)	1 (2,3)	**0,014**	0 (0)	2 (5,7)	0,173
Clavien-Dindo IV°	7 (8,6)	1 (1,3)	**0,034**	4 (8,2)	1 (2,3)	0,220	3 (9,4)	0 (0)	0,066
Clavien-Dindo V°	1 (1,2)	0 (0)	0,326	1 (2,0)	0 (0)	0,349	0 (0)	0 (0)	–
WOMAC, Med (IQA)									
Präoperativ	59,0 (24)	62,0 (28)	0,999	64,5 (23)	69,0 (24)	0,500	57,0 (17)	56,0 (34)	0,584
4 Wochen postoperativ	24,0 (23)	25,5 (24)	0,624	22,5 (23)	27,0 (29)	0,737	28,5 (32)	29,0 (18)	0,259
3 Monate postoperativ	15,0 (21)	15,0 (22)	0,969	12,0 (15)	11,0 (20)	0,907	27,0 (24)	20,0 (18)	0,423
EQ-5D-VAS, Med (IQA)									
Präoperativ	50,0 (28)	60,0 (30)	0,172	50,0 (36)	50,0 (34)	0,840	50,0 (29)	60,0 (30)	**0,028**
4 Wochen postoperativ	67,5 (30)	65,0 (30)	0,593	70,0 (33)	70,0 (23)	0,925	60,0 (21)	65,0 (30)	0,264
3 Monate postoperativ	70,0 (25)	75,0 (30)	0,093	70,0 (25)	80,0 (30)	0,448	65,0 (30)	75,0 (30)	0,068

*SPPB* Short Physical Performance Battery, *WOMAC* Western Ontario and McMaster Universities Osteoarthritis Index, *EQ-5D-VAS* Visuelle Analogskala, *Med* Median, *IQA* Interquartilsabstand, *n* Anzahl, *SOG* Spezielle Orthopädische Geriatrie

Die gefetteten *p*-Werte sind signifikant (<0,05)

**Figure 3:**
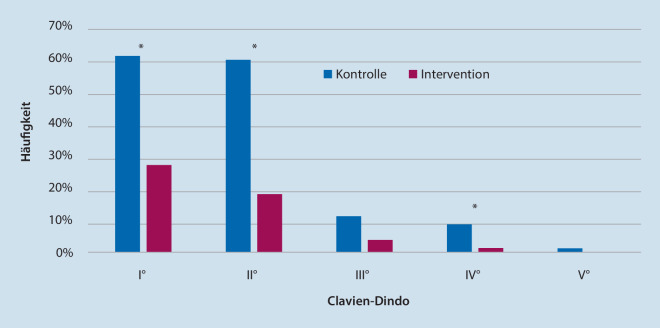

Das Risiko für erst- und zweitgradige Komplikationen war in der Interventionsgruppe gegenüber der Kontrollgruppe um 34 bzw. 41 % signifikant reduziert (*p* < 0,01, Abb. 3). Bezüglich drittgradiger Komplikationen zeigte sich mit 11 % vs. 4 % ein deutlicher Trend zu einer Komplikationsreduktion in der Interventionsgruppe, statistisch war dies jedoch nicht signifikant. Bei viertgradigen Komplikationen zeigte sich mit 9 % vs. 1 % eine signifikante Häufung in der Kontrollgruppe (*p* < 0,04, Abb. 3). Die Detailauswertung der Major-Komplikationen zeigte für III° in der Kontrollgruppe vier intraoperative Frakturen (3 Tochanterabrissfrakturen, eine Kalkarfraktur), eine postoperative Sinterung mit Kalkarfraktur, 2 tiefe Frühinfektionen, 2 infizierte epifasziale Hämatome und ein Sturzereignis mit Rissquetschwunde im Gesicht. In der Interventionsgruppe kam es zu einer intraoperativen Trochanterfraktur, einem postoperativen Sturz mit pertrochantärer Femurfraktur und einer traumatischen Wunddehiszenz mit konsekutivem Frühinfekt. An viertgradigen Komplikationen traten in der Kontrollgruppe 3‑mal schwere postoperative Delire, 2 instabile Tachyarrhythmien und 2 akute Koronarsyndrome auf. In der Interventionsgruppe kam es zu einem Grand-Mal-Anfall. Infolge eines Apoplex trat in der Kontrollgruppe ein Sterbefall auf (Tab. 2).

**Figure 4:**
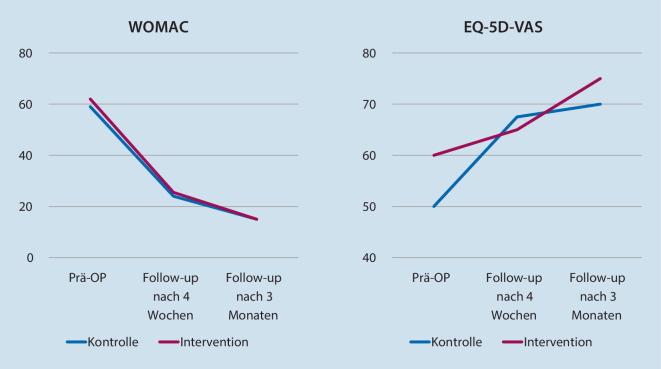

Die Auswertung der WOMAC- und EQ-5D-VAS-Daten zeigte für Kontroll- und Interventionsgruppe 4 Wochen und 3 Monate postoperativ keine relevanten Unterschiede (Tab. 2). In beiden Gruppen kam es zu einer klinisch relevanten Verbesserung von Gelenkfunktion und gesundheitsbezogener Lebensqualität (Abb. 4).

## Diskussion

### Mobilität

Die primäre Outcomevariable Mobilität, gemessen anhand der SPPB, nahm sowohl in der Interventionsgruppe als auch in der Kontrollgruppe in der frühen postoperativen Phase leicht ab, erholte sich bereits in der ersten Woche auf das Niveau der Ausgangswerte und war 4 und 12 Wochen postoperativ schließlich besser als vor der Operation. Patienten der Interventionsgruppe wiesen peri- und postinterventionell zu allen Erfassungszeitpunkten eine bessere Mobilität auf als Patienten der Kontrollgruppe. So zeigte sich 3 Tage, 7 Tage, 4 Wochen und 12 Wochen postoperativ eine um 1–2 Punkte höhere SPPB.

Aufgrund der einfachen Anwendung und hervorragenden prognostischen Validität gilt die SPPB als Goldstandard im Mobilitätsassessment bei geriatrischen Patienten [16]. Mehrere Validierungsstudien zeigten eine Korrelation der SPPB mit Frailty, Sarkopenie, postoperativen Komplikationen und Mortalität [4, 8, 25]. Eine Verbesserung um 0,5 Punkte gilt bereits als klinisch relevant, eine Verbesserung um mehr als einen Punkt wird als klinisch bedeutungsvoll angesehen [26].

Vergleichsdaten zur SPPB bei geriatrischen Patienten nach primärendoprothetischer Versorgung im Rahmen einer integrierten orthogeriatrischen Versorgung sind in der Literatur kaum zu finden. Przkora et al. beschreiben in ihrer Machbarkeitsstudie zum Mobilitätsassessment bei geriatrischen Patienten 2 Wochen nach elektiver Knie-TEP-Implantation eine Verschlechterung der SPPB um 4,8 Punkte im Vergleich zu den präoperativen Ausgangswerten [28]. Die initiale Verschlechterung der Mobilität in der Frühphase nach Gelenkersatz konnte in der aktuellen Studie nachvollzogen werden, jedoch fiel diese deutlich geringer aus und war bereits eine Woche postoperativ nicht mehr vorhanden (Abb. 2), was vermutlich auf die schlechtere präoperative Mobilität in unseren Studiengruppen zurückzuführen ist (7 vs. 10,8).

Die Datenlage unter Einbeziehung alternativer Mobilitätsassessments (Timed-Up-And-Go-Test, Gait Speed) nach elektivem Gelenkersatz ist etwas besser, jedoch kontrovers. In mehreren prospektiv-randomisierten Studien wurde die Mobilität nach Hüft-TEP-Implantation im Fast-Track-Schema gegenüber einer Kontrollgruppe mit Standardversorgung verglichen, wobei sich insbesondere in der frühen postoperativen Phase eine deutlich bessere Mobilität in den Fast-Track-Gruppen zeigte [6, 17]. Eine weitere prospektiv-randomisierte Studie und eine Metaanalyse kamen zu dem Ergebnis, dass die Mobilität ab 4 Wochen postoperativ zwischen Fast-Track-Gruppen und Kontrollgruppen vergleichbar ist [1, 3]. Sehr gute Evidenz existiert für interdisziplinäre orthogeriatrische Modelle in der Versorgung hüftgelenksnaher Frakturen. Hier zeigte sich eine deutliche Überlegenheit bezüglich Mobilität und funktioneller Aspekte im Vergleich zur traumatologischen Standardversorgung, was mittlerweile zur flächendeckenden Etablierung alterstraumatologischer Zentren geführt hat [23].

### Postoperative Komplikationen

Als sekundäre Outcomevariable wurden postoperative Komplikationen ausgewertet. Die vergleichsweise hohe Komplikationsrate von bis zu 60 % für Clavien-Dindo I° und II° überraschen auf den ersten Blick, stehen jedoch durchaus im Einklang mit der Literatur. So zeigten retrospektive Analysen bei geriatrischen Patienten vergleichbare Komplikationsraten nach elektiver Hüft- oder Knie-TEP-Implantation [22]. Im Vergleich zwischen Kontroll- und Interventionsgruppe zeigte sich bei den Minor-Komplikationen (Clavien-Dindo I° und II°) eine relative Risikoreduktion um 55 bzw. 70 % in der Interventionsgruppe, was einer „number needed to treat“ von 2–3 entspricht. Bei den Major-Komplikationen mit chirurgischem Interventionsbedarf (Clavien-Dindo III°) und intensivmedizinischer Behandlungsnotwendigkeit (Clavien-Dindo IV°) zeigte sich – auch wenn statistisch nur teilweise signifikant – ebenfalls eine relative Risikoreduktion von 66 bzw. 84 % in der Interventionsgruppe. Ein verringertes Risiko für postoperative Komplikationen durch orthogeriatrisches Co-Management bei Gelenkersatz wurde in der Literatur bereits von mehreren Autoren beschrieben [9, 12, 14, 24].

Im Hinblick auf die Detailauswertung der Komplikationen mit chirurgischem Interventionsbedarf muss auf die niedrigere Anzahl an intraoperativen Frakturen in der Interventionsgruppe hingewiesen werden (4 vs. 1). Bei identischer Femurpräparationstechnik in beiden Gruppen ist dieser Unterschied wohl am ehesten auf eine zufällige Häufung in der Kontrollgruppe zurückzuführen. Das Ausbleiben schwerer postoperativer Delire und intensivpflichtiger kardialer Ereignisse in der Interventionsgruppe könnte jedoch durchaus auf einen Effekt des internistisch-geriatrischen Co-Managements hinweisen.

### „Patient reported outcome“

Um den von Patienten subjektiv wahrgenommenen Therapieeffekt zu erfassen wurden „patient-reported outcome measures“ (PROM) ausgewertet. Dabei zeigte sich 4 Wochen und 3 Monate postoperativ unabhängig von Kontroll- und Interventionsgruppe eine deutliche Verbesserung sowohl der Gelenkfunktion (WOMAC) als auch der gesundheitsbezogenen Lebensqualität (EQ-5D-VAS), was im Einklang mit den Daten diverser Prothesenregister steht [10]. Das Verbesserungsausmaß der PROM war in der Interventionsgruppe, trotz objektiv besserer Mobilität und geringerer Komplikationsrate, vergleichbar zur Kontrollgruppe. In der Literatur finden sich mehrere Hinweise, dass sich eine objektive Funktionsverbesserung und/oder Komplikationsreduktion nach Gelenkersatz nicht unbedingt in verbesserten PROM widerspiegelt [6, 20]. Möglicherweise sind die verwendeten Fragebögen zu unscharf, um den klinisch evidenten Vorteil der Intervention hier herauszuarbeiten. Zudem wurden Verständnisprobleme bei der Verwendung der teils komplexen Fragebögen, insbesondere bei kognitiv beeinträchtigen geriatrischen Patienten beobachtet.

### Limitationen und Stärken

Bei der Interpretation der vorliegenden Daten muss beachtet werden, dass es sich um eine Zwischenanalyse handelt. Von den geplanten 139 Patienten pro Studiengruppe absolvierten bis zum Zeitpunkt der Zwischenanalyse etwas mehr als die Hälfte das letzte Follow up (Abb. 1). Obwohl in den Daten bereits jetzt signifikante Unterschiede zu erheben sind, können sich bis zum Studienabschluss noch Abweichungen ergeben. Eine weitere Limitation dieser Studie ist die nur teilweise mögliche Verblindung. Obwohl das Ergebnis der Randomisierung nicht kommuniziert wird, können Teilnehmer durch die studienärztliche Aufklärung im Rahmen des Studieneinschlusses Rückschlusse auf ihre Zugehörigkeit zu Kontroll- oder Interventionsgruppe ziehen, wodurch ein gewisser Detection-Bias in Betracht zu ziehen ist. Als multidisziplinäre Intervention ist die Wirksamkeit der integrierten orthogeriatrischen Versorgung von der Verfügbarkeit, Qualität und Kooperation der beteiligten Behandler abhängig. Die Überlegenheit gegenüber der Standardversorgung sollte deshalb im multizentrischen Setting bestätigt werden.

Trotz dieser Limitationen wird durch das GBA-Projekt „Spezielle Orthopädische Geriatrie“ (SOG) erstmalig der positive Effekt einer integrierten orthogeriatrischen Versorgung auf Mobilität und Komplikationsrisiko nach elektiver Hüft- oder Knie-TEP-Implantation im Rahmen einer kontrollierten prospektiv-randomisierten Studie mit bis dato 174 geriatrischen Studienteilnehmern demonstriert. Dem Beispiel der Alterstraumatologie folgend, könnte dies ein weiterer Schritt hin zu einer verbesserten Versorgung geriatrischer Patienten in der elektiven orthopädischen Chirurgie sein.

## Ausblick

Die integrierte orthogeriatrische Versorgung scheint im Vergleich zur Standardversorgung in der Hüft- und Kniegelenkendoprothetik Vorteile bezüglich Mobilität und Komplikationsrate zu bieten. Integrierte orthogeriatrische Modelle wie die Spezielle Orthopädische Geriatrie könnten zukünftig die Versorgung geriatrischer Patienten in der elektiven orthopädischen Chirurgie verbessern und vor allem sicherer gestalten.

## Fazit für die Praxis

Durch eine integrierte orthogeriatrische Versorgung in der elektiven Hüft- und Kniegelenkendoprothetik kann

- die postoperative Mobilität verbessert und
- das Komplikationsrisiko reduziert werden.

## Abkürzungen

BfArM: Bundesinstitut für Arzneimittel und Medizinprodukte
BMI: Body-Mass-Index
CCI: Charlson-Komorbiditätsindex
DLR: Deutsches Zentrum für Luft- und Raumfahrt
DRKS: Deutsches Register Klinischer Studien
G‑BA: Gemeinsamer Bundesausschuss
GDS: Geriatrische Depressionsskala
GFK: Geriatrische frührehabilitative Komplexbehandlung
IADL: Instrumentelle Aktivitäten des täglichen Lebens
IQA: Interquartilsabstand
MMST: Mini-Mental-Status-Test
PBM: „Patient blood management“
PROM: „Patient-reported outcome measures“
RCT: Randomisierte kontrollierte Studie
SOG: Spezielle Orthopädische Geriatrie
SPPB: Short Physical Performance Battery
TEP: Totalendoprothese
UN: United Nations (Vereinte Nationen)
VAS: Visuelle Analogskala
WHO: World Health Organization
WOMAC: Western Ontario and McMaster Universities Osteoarthritis Index
